# Vitamin D for primary dysmenorrhea and endometriosis-related pain – A systematic review of registered RCTs

**DOI:** 10.1371/journal.pone.0321393

**Published:** 2025-04-21

**Authors:** Iris Wenyu Zhou, Anthony Lin Zhang, Miranda Sin-Man Tsang, Charlie C. Xue

**Affiliations:** The China-Australia International Research Centre for Chinese Medicine, School of Health and Biomedical Sciences, STEM College, RMIT University, Bundoora, Victoria, Australia; Mater Olbia Hospital, ITALY

## Abstract

**Objective:**

This systematic review investigates the potential role of vitamin D supplement in alleviating pain associated with primary dysmenorrhea or endometriosis by analysing registered randomised controlled trials (RCTs).

**Methods:**

We comprehensively searched the WHO International Clinical Trials Registry Platform to identify registered RCTs that assessed the effects of vitamin D supplement on pain outcomes in people with primary dysmenorrhea or endometriosis. The primary outcomes of interest were pain severity/intensity, pain duration, pain medication usage and pain-related outcome measurements.

**Results:**

Seven registered RCTs were included in this systematic review. These RCTs had diverse doses and treatment durations of vitamin D supplement used as intervention. They also had either high or some concerns of risk of bias, according to Cochrane risk of bias version 2 assessment. Substantial heterogeneities were generally observed across the seven RCTs. When measured using a validated tool visual analogue scale (VAS), pain severity was significantly lower at the end of treatment when compared with placebo (mean difference MD –1.12, 95% confidence interval [–2.16, –0.07], I^2^=81%, 5 studies, n = 308). This significant difference was observed in the primary dysmenorrhea RCTs, but not the endometriosis RCTs.

**Conclusion:**

This systematic review identified vitamin D may reduce pain associated with primary dysmenorrhea, though a low certainty of evidence was available. Future studies that use standardised doses and treatment duration in accordance with the latest clinical practice guidelines are needed to explore any potential benefits vitamin D may have for people with these conditions.

## Background

Primary dysmenorrhea, a common gynaecological condition, refers to painful menstrual cramps that occur in the absence of any underlying pelvic pathology [1]. It typically occurs shortly before or during menstruation and involves pelvic pain. A 2014 review on longitudinal data revealed that primary dysmenorrhea affects up to 90% women of reproductive age, and 2–29% of women of this age group suffer severe pain [2].

Endometriosis is the most common cause of secondary dysmenorrhea. It is a chronic condition characterised by the presence of endometrial-like tissue outside the uterus, commonly found in the pelvic cavity [1]. It is a complex disorder that affects approximately 10–15% of women of reproductive age, and is a leading cause of chronic pelvic pain and infertility [3].

The exact cause of primary dysmenorrhea remains unclear, but it is believed to involve increased production of prostaglandins, hormone-like substances that play a role in uterine contractions [4]. During menstruation, the uterus contracts to expel the endometrial lining. In women with primary dysmenorrhea, higher levels of prostaglandins are produced, leading to increased uterine contractions and stronger pain signals [5]. These contractions can restrict blood flow to the uterus, leading to ischemia and pain [6]. In addition to prostaglandins, other factors, such as genetic predisposition, hormonal fluctuations and psychological factors, may contribute to the development and severity of primary dysmenorrhea [2,7]. Risk factors for primary dysmenorrhea include earlier age at menarche, heavy menstrual flow, and smoking [8].

The exact cause of endometriosis is also not fully understood, but several mechanisms have been proposed. One widely accepted mechanism is retrograde menstruation, where menstrual blood containing endometrial cells flows backward through the fallopian tubes into the pelvic cavity instead of exiting the body. These displaced endometrial cells can adhere to and invade the pelvic organs, forming endometriotic lesions. However, retrograde menstruation alone does not fully explain the development of endometriosis, as many women experience retrograde menstruation without developing the condition [9].

Other proposed mechanisms include immune dysfunction, genetic factors, and hormonal imbalances. Normally, immune cells (e.g., macrophages, natural killer cells and cytotoxic T cells) surveys and scavenges menstrual debris entering the peritoneal cavity without eliciting a significant inflammatory reaction [10]. However, impaired surveillance and clearance of the displaced endometrium may permit viable endometrial cells to attach and grow ectopically[10]. Meanwhile, DNA hypermethylation at the promoter of the progesterone receptor isoform B (PR-B) has been shown to contribute to the onset and progression of endometriosis[11], suggesting the involvement of epigenetic mechanisms, which refer to modifications in gene expression without changes to the underlying DNA sequence[11]. In endometriosis, a deficit in progesterone signalling or an imbalance between estrogen and progesterone leads to uncontrolled growth of endometrial tissue outside the uterus. This hormonal imbalance may further intensify immune dysfunction, creating a positive feedback loop[12,13].

In Western medicine, primary dysmenorrhea and endometriosis are primarily managed through pharmaceutical interventions. For primary dysmenorrhea, nonsteroidal anti-inflammatory drugs (NSAIDs) are prescribed to mitigate pain and inflammation associated with menstruation. For endometriosis, hormonal therapies, such as birth control pills, gonadotropin-releasing hormone agonists and progestins, are prescribed to regulate the menstrual cycle and alleviate symptoms. Surgical options, such as laparoscopic excision of endometrial tissue, may be considered for severe cases. While these interventions can provide relief, they are not without unwanted effects [14–17]. Hormonal therapies may cause adverse effects and are not suitable for all patients, and surgical procedures carry inherent risks.

Recently, interest in the role of supplement products, such as vitamin D, in managing these two conditions has increased. Some studies [18,19] suggest vitamin D deficiency may be linked to increased severity of symptoms, and supplement to pharmaceutical treatments may be beneficial to patients. Therefore, it is critical to understand the potential role vitamin D may play in managing these two conditions.

### Vitamin D’s effect on primary dysmenorrhea- and endometriosis-related pain

Vitamin D is a fat-soluble vitamin that is synthesised in the skin after exposure to sunlight and can be obtained through diet and supplement. It has a vital role in bone health, immune function and hormonal regulation. In particular, vitamin D’s anti-inflammatory and immunomodulatory properties make it an intriguing candidate for further study related to primary dysmenorrhea and endometriosis.

Exploring the potential impact of vitamin D on prostaglandin regulation in people with primary dysmenorrhea is of great interest. Prostaglandins play a role in uterine contractions and pain sensation during menstruation. If vitamin D can modulate prostaglandin release or their effects on the uterus, their use could offer a novel approach to managing primary dysmenorrhea-related pain, potentially reducing reliance on pain medication and improving the overall wellbeing of people with this condition [20].

Chronic inflammation is a key feature of endometriosis, contributing to endometrial lesion growth and progression. Hormonal imbalances also play a crucial role in the development and progression of endometriosis [21]. If vitamin D supplement can help reduce inflammation and restore hormonal balance, it may have a beneficial effect on endometrial tissue growth and lesion formation. This could improve symptom management and potentially even prevent endometriosis onset in susceptible individuals.

Several systematic reviews have explored the potential effects of vitamin D on primary dysmenorrhea [22] and endometriosis [23]. Due to the heterogeneity and diversity of available studies (human clinical trials, *in-vivo* and *in-vitro* studies), a clear conclusion on the effect of vitamin D on endometriosis is not available [23]. However, pooled data from nine RCTs showed vitamin D supplement can reduce dysmenorrhea-related pain among women with primary dysmenorrhea and vitamin D deficiency [22].

If vitamin D supplement is effective for reducing pain and inflammation associated with primary dysmenorrhea and/or endometriosis, it could offer a promising adjunct therapy to existing treatments or even a preventive therapy. Therefore, this systematic review explores the efficacy of vitamin D on these two conditions, with a specific focus on pain outcome measures.

## Methodology

The protocol for this systematic review was registered with PROSPERO (CRD42023470800). The review was prepared in accordance with the PRISMA guidelines and Cochrane systematic review methodology.

### Search strategy and identification of studies

Clinical trials were identified from the World Health Organisation International Clinical Trials Registry Platform (ICTRP) on 24 June 2023. Results of each search term (vitamin D, endometriosis, primary dysmenorrhea, and their synonyms), exported from ICTRP in XML format, were imported in MS Excel and pooled. Duplicates and non-interventional studies were removed from the results. Registered randomised controlled trials (RCTs) with results published in peer-reviewed full-text articles were screened independently by two researchers based on the characteristics of participants and intervention of the RCT to determine eligibility for inclusion in this review. The search process was recorded in a PRIMSA 2020 diagram template, and a Shiny App diagram generator was used to produce the figures.

## Inclusion and exclusion criteria

### Study design

Prospective RCTs published in English, with no restrictions on publication type were included. Retrospective studies were excluded.

### Participants

Female adults diagnosed with primary dysmenorrhea or endometriosis, with or without a history of surgical intervention for their condition, were included. RCTs investigating patients diagnosed with other unrelated conditions or diseases were excluded.

### Test interventions

Test interventions encompassed orally administered vitamin D supplement. These interventions could take various forms, including liquids, granules, capsules or pills. RCTs where vitamin D was administered as the single intervention for primary dysmenorrhea or endometriosis were included. RCTs where vitamin D tested as one of multiple supplements were excluded.

## Control interventions

### Control interventions were placebo or no treatment

#### Outcome measures.

Outcome measures comprised assessments related to pain and its impact. These included, but were not limited to, pain, including its severity and impact on quality of life. RCTs that did not assess pain-related outcomes were excluded.

#### Risk of bias assessment.

Risk of bias in each outcome measure was assessed using the Cochrane Risk of Bias 2 (RoB2) tool by two independent reviewers (IWZ and MSMT). Consensus was reached via discussion whenever there was disagreement in judgment and, mediated by a third reviewer as necessary (ALZ). Traffic light plots were generated using robvis visualisation tool. Potential publication bias was not assessed because less than 10 eligible studies were available.

#### Meta-analysis.

Meta-analysis was conducted in Review Manager (version 5.4.1). Mean difference (MD), risk ratio (RR) and 95% confidence intervals (CI) were calculated. Heterogeneity was measured using the I^2^ statistic. Conservative random-effect models were used due to likely heterogeneity in study populations and methods. Baseline scores were compared between groups to determine baseline comparability. Sensitivity analyses were planned to explore any effects of baseline imbalances, study duration and/or intervention dose. The certainty of the evidence was assessed using Grading of Recommendations Assessment, Development and Evaluation (GRADE).

## Results

### Literature search results

**Fig 1:**
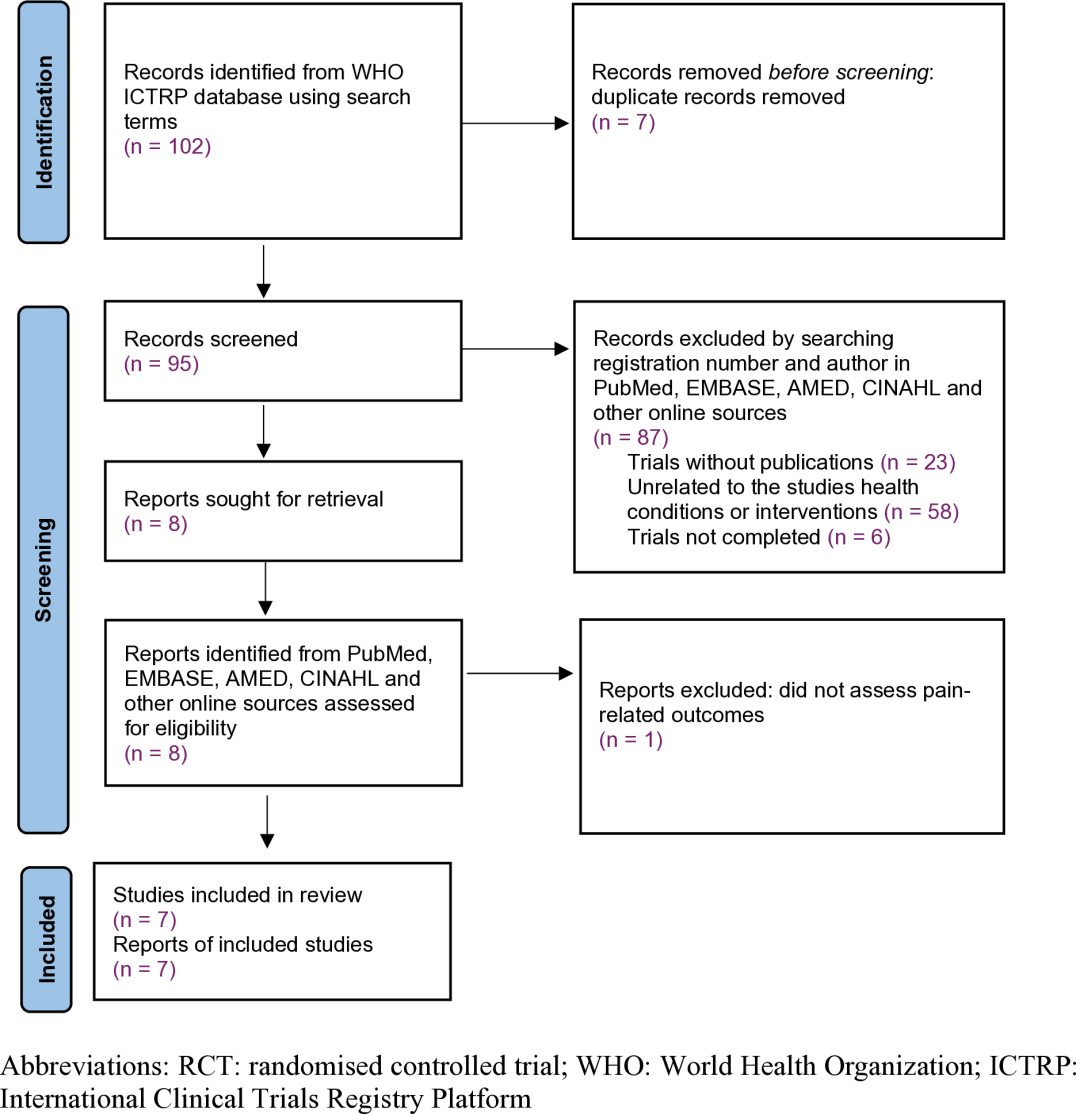
Flow diagram of the search and study selection process.

After duplicates were removed, 95 registered RCTs remained. Trial registration number and authors were used as search terms to explore whether the trial has been published. Based on the search results from four main English databases (PubMed, Embase, AMED and CINAHL) and Google scholar, trials that were not completed (n = 6), trials without publications (n = 23), and trials that were unrelated to the studied health conditions or interventions (n = 58) were excluded. Eight full-text papers relating to vitamin D were obtained for further assessment against the inclusion and exclusion criteria (see S1 Table 1: numbered table of all studies identified in the literature search). One RCT [24] did not assess pain-related outcomes was excluded. The remaining seven RCTs [25–31] satisfied the selection criteria and were included in this review (Fig 1).

### Participants

The seven RCTs enrolled 510 participants (primary dysmenorrhea, four RCTs, T = 182, C = 180; endometriosis, three RCTs, T = 76, C = 72). Age ranged from 18–50 years for primary dysmenorrhea and, 12–40 years for endometriosis (Table 1).

Two of the three endometriosis RCTs [25,28] were conducted in Iran with patients admitted to hospital settings, and the third endometriosis RCT [29] involved nonpregnant women diagnosed with endometriosis in a hospital clinic in the USA. In this RCT [29], only people with vitamin D levels of 25[OH]D <100 ng/mL were included. Two RCTs [25,29] included only people with a Visual Analogue Scale (VAS) score 3 or higher.

The four RCTs on primary dysmenorrhea pain were all conducted in Iran. Two [26,31] enlisted university students with primary dysmenorrhea and vitamin D deficiency. One of these RCTs [26] defined vitamin D deficiency as 25[OH]D ≤30 ng/mL) while the other [31] defined vitamin D deficiency as 25[OH]D <30 ng/mL, according to the Endocrine Society Clinical Practice Guideline[32]. The third RCT [30] recruited university students with primary dysmenorrhea, and the fourth RCT [27] included women with primary dysmenorrhea, without specifying the setting.

One RCT (30) included people with moderate-to-severe primary dysmenorrhea. Another [31] included people with a numerical rating scale (NRS) ≥4. The other two RCTs [26,27] did not specify the severity of pain as an inclusion criteria.

**Table 1:** Characteristics of all RCTs.

Author name; year of publication; country	Setting; participants number (T, C); age range	Treatment dose; duration; follow-up period	Control	Pain-related outcome measures; outcome tool; measurement timepoint
***Primary dysmenorrhea***				
Amzajerdi; 2023; Iran	University students with primary dysmenorrhea and vitamin D deficiency (25[OH]D ≤30 ng/mL); (39, 39); 18–25	300,000 IU vitamin D taken as 50,000 IU, two tablets, every 8 h, for one day, 5 days before the putative beginning of menstrual cycle; once off; 2 months’ follow up	Placebo (paraffin)	Menstruation pain; VAS; second month post intervention Plus perception of menstrual pain severity and its effect on daily activities; VMS; second month post intervention
Behrouzi; 2023; Iran	People with primary dysmenorrhea; (25, 25); 18–50	vitamin D 1000 IU daily; 2 months; no follow up	Placebo (starch)	Severity/intensity of menstrual pain; end of 2 months of treatment; VAS Pain duration (days) from the onset of dysmenorrhea; CMSS end of 2 months of treatment
Pakniat; 2019; Iran	University students with moderate-to-severe primary dysmenorrhea; (60,60); 18–25	vitamin D 1000 mg tablet daily for up to 3 days after the onset of menstrual flow plus mefenamic acid 250 mg twice daily; 5 days, 2 months follow up	Placebo (n.s.) plus mefenamic acid 250 mg twice daily	Severity of pain at 2^nd^ month post-intervention: VAS; and VAS score change from pre-intervention to 2^nd^ month post-intervention
Rahnemaei; 2021; Iran	University students with primary dysmenorrhea (NRS ≥4) and vitamin D deficiency (25[OH]D <30 ng/mL); (58, 56); 18–32	vitamin D 3 50,000 IU weekly; 8 weeks, no follow up	Placebo (inert oil-like corn)	Pain measured at 8 weeks after intervention: Pain intensity, 11-point NRS; Pain duration, days with pain in each menstrual cycle; pain medication, number of pain-relief medication per day during menstrual cycle.
***Endometriosis***				
Almassinokiani; 2016; Iran	Hospital patients with endometriosis and VAS ≥3; (19, 20); 15–40	vitamin D3 50,000 IU weekly; 12 weeks; 4 weeks’ follow up	Placebo (n.s.)	Severity of dysmenorrhea pain; VAS; 4 weeks after treatment
Mehdizadehkashi; 2021; Iran	Hospital patients with endometriosis; (30, 30); 18–40	vitamin D 50,000 IU every 2 weeks; 12 weeks; no follow up	Placebo (n.s.)	Dysmenorrhea pain; clinical symptoms; score change between baseline and after treatment at week 12
Nodler; 2020; USA	Nonpregnant females with endometriosis from a hospital clinic and vitamin D level of 25[OH]D <100 ng/mL and VAS≥3; (27, 22); 12–25	vitamin D3 2000 IU daily; 6 months; no follow up	Placebo (inert lactose powder)	Worst pain in the past month; pain sensitivity measured by VAS; Perception measured by catastrophic thinking; mean amount of pain medication (non-narcotic pain tablets) taken per week; end of 6 months of treatment

Abbreviations: T, treatment/intervention; C, control; n.s., not specified; VAS, Visual Analogue Scale; SF12, Short Form 12; VMS, verbal multidimensional scoring system; NRS, numerical rating scale; CMSS, Cox Menstrual Symptom Scale.

VAS: 0–10, 0 being no pain, 10 being the worst pain; NRS: 0–10, 0 indicating no pain, 5 moderate pain and 10 the worst possible pain; VMS: 0–3, 0 = none, 1 = mild, 2 = moderate and 3 = severe; CMSS: 0–4, 0 = none, 1 = lasting 0–3 h, 2 = lasting 3–7 h, 3 = lasting 7–24 h and 4 = lasting > 24 h; Catastrophic thinking (validated measure of pain sensitivity): 0–52, higher scores indicate a greater amount of catastrophising.

Date of data extraction: Oct 2023; name of data extractors: IWZ and MSMT.

### Interventions

Three RCTs, two on endometriosis [25,29] and one on primary dysmenorrhea [31], explicitly prescribed vitamin D3 (cholecalciferol). The remaining four RCTs did not specify the vitamin D subtype.

Treatment doses and durations varied significantly among studies. In two endometriosis studies, 50,000 IU either weekly [25] or every two weeks [28] was prescribed for 12 weeks. In the third endometriosis study [29], 2000 IU daily was prescribed for six months.

For the two RCTs on primary dysmenorrhea with vitamin D deficiency [26,31], participants were given either 50,000 IU weekly for 8 weeks [31] or a total of 300,000 IU (50,000 IU, two tablets, every eight hours), five days before the putative beginning of menstrual cycle [26].

For the other two RCTs on primary dysmenorrhea that didn’t specify vitamin D deficiency as an inclusion criteria [27,30], participants were given either 1000 IU daily for two months [27] or 1000 mg daily for up to three days after the onset of menstrual flow for five days [30]. In this latter RCT [30], mefenamic acid 250 mg was also given to participants in both placebo and intervention groups twice daily.

### Comparison

All seven RCTs used a placebo as control in their study design, with detail of the placebo specified in four studies [26,27,29,31]. The manufacturing company that produced the placebo was mentioned in all but one RCT [27].

### Outcome measurements

Four kinds of pain-related measures were reported in these seven trials.

Pain severity/intensity was measured using a validated tool [33] – VAS in five RCTs [25–27,29,30], an 11-point NRS in one RCT [31] or clinical symptoms including dysmenorrhea score change between baseline and after treatment in the other RCT [28].

Pain duration was reported in two RCTs [27,31]. One RCT [27] used a 5-point Cox Menstrual Symptom Scale (CMSS) and the other RCT [31] measured days with pain in each menstrual cycle.

Two RCTs [29,31] measured pain medication use. One RCT [29] calculated the mean amount of pain medication (non-narcotic pain tablets) taken per week, while the other RCT [31] collected data on the number of pain relief medications taken per day during the menstrual cycle.

One RCT [29] measured pain sensitivity using a validated tool – the catastrophic thinking scale.

The RCT with the highest single vitamin D dose [26] measured perception of menstrual pain severity and its effect on daily activities using the verbal multidimensional scoring system (VMS).

### Risk of bias

**Fig 2:**
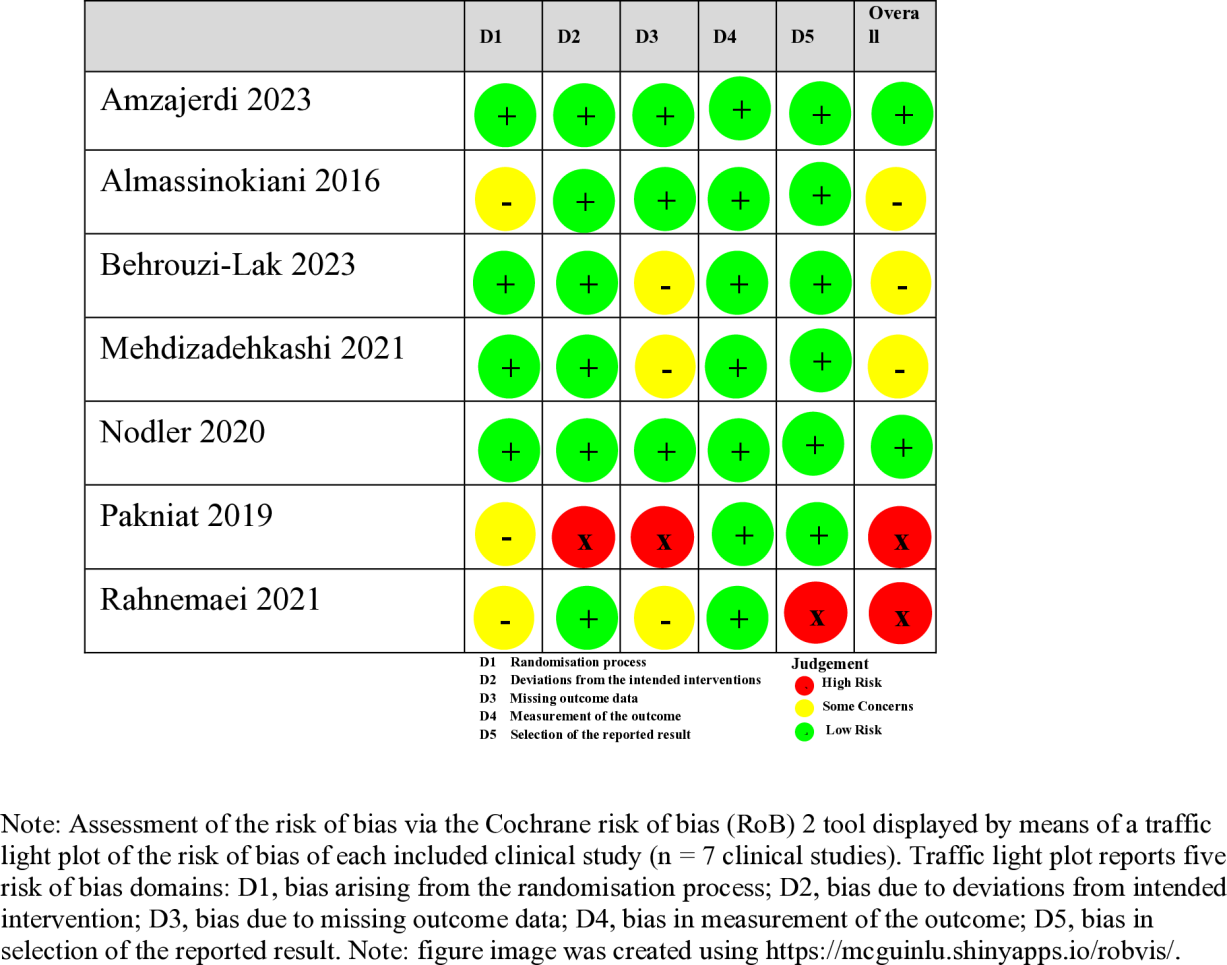
Risk of bias of included RCTs.

Risk of bias assessments were conducted with RoB2 (see Fig. 2). All seven RCTs described their randomisation method. Three RCTs used permuted block randomisation [29–31], two used computer-based randomisation [27, 28], one used a random number table [26] and one used simple randomisation [25]. The allocation sequence was concealed in five RCTs [26–29,31], but one of them [31] reported baseline differences in an outcome measurement, so was assessed as ‘some concerns’ for the randomisation process. In addition, information on sequence concealment was not adequate/available in two other RCTs [25,30], so risk of bias was assessed as ‘some concerns’ for these two studies. The other four studies [26–29] were assessed as low risk for this domain.

Six studies were double-blinded [25–29,31] and it appeared the participants and people delivering the intervention were not aware of the assignment, so ‘deviations from the intended interventions’ were considered as low risk. One single-blinded (of participants) RCT [30] was ranked as high risk for this domain, because the people delivering the intervention were aware of the intervention and whether participants were adhered to the intervention was not analysed.

For ‘missing outcome data’, one RCT [30] was assessed as high risk because reasons of lost to follow up was not stated and it is possible that outcome data missing was influenced by lost to follow up. Three RCTs [27,28,31] were assessed as ‘some concerns’ because reasons of lost to follow up was not stated. Three RCTs were assessed as low risk for this domain, because outcomes were available for nearly all participants [25] or reasons of lost to follow up was stated and were unrelated to the outcome [26,29].

All RCTs were assessed as low risk for ‘measurement of the outcome’, because outcome assessors were not aware of the intervention participants received.

One RCT [31] was assessed as high risk for ‘selection of the reported result’ because outcome measures were not consistent between the registration and published paper. The remaining RCTs were assessed as low risk for this domain, because information in their registration was consistent with their reporting, although none of them had a published trial protocol.

## Treatment outcomes

### Pain severity/intensity

**Fig 3:**
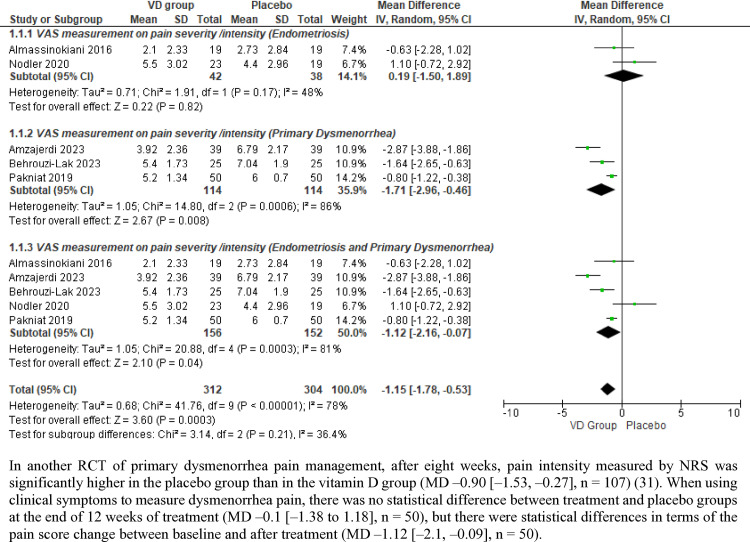
Forest plots of VAS measurements on pain severity or intensity.

When measured by VAS (in five RCTs [25–27,29,30]), pain severity was significantly lower at the end of treatment for people prescribed with vitamin D than those prescribed with placebo (MD –1.12[–2.16, –0.07], I^2^ = 81%, n = 308) (Fig 3). This between-group difference was also observed among the three RCTs on primary dysmenorrhea [26,27,30] (MD –1.71 [–2.96, –0.46], I^2^ = 86%, n = 228) but not in the two endometriosis RCTs [25,29] (MD 0.19 [–1.50, 1.89], I^2^ = 48%, n = 80).

### Pain duration

One RCT used a 5-point scale CMSS to evaluate pain duration in 50 individuals with primary dysmenorrhea [27]. Two months after receiving intervention (at the third menstrual cycle), pain duration (in days) in the vitamin D group was significantly lower than that in the placebo group (MD –1.56 [–2.06, –1.06], n = 50). Pain duration was also measured in another RCT on primary dysmenorrhea [31], but reported data were in median and 25%, 75% interquartile range. Meta-analysis was not done.

### Pain medication use

One endometriosis RCT reported on pain medication use (non-narcotic pain tablets) per week [29]. The RCT reported similar medication use at the end of six months of treatment (MD –0.1 [–3.39, 3.19], n = 43). Information about the amount of pain relief medication used per day was collected in a primary dysmenorrhea RCT [31], but reported data were in median and 25%, 75% interquartile range. Meta-analysis was not done.

### Other pain measures

A primary dysmenorrhea RCT [26] measured perception of pain and its effect on daily activities using VMS. The baseline measurement was the same (2.0), but there was a notable lower score in participants in the vitamin D group compared to the placebo group. The between group mean difference was (MD –1.11 [–1.37 to –0.85], n = 78).

Catastrophic thinking for pain sensitivity was measured in an endometriosis study [29], but no between-group difference was reported (MD 1.20 [–4.21, 6.61], n = 42).

### Adverse events

One RCT [31] reported no adverse event in the vitamin D or placebo groups. Another RCT reported mild nausea (n = 2) in the placebo group and no adverse event in the vitamin D group [30]. The other studies did not report any adverse event information.

### Certainty of evidence

The certainty of evidence was assessed according to the GRADE system on the four most relevant pain measurements. As showed in Table 2, the certainty of evidence was low for VAS and NRS, and moderate for CMSS and pain medication use.

**Table 2:** Summary of evidence certainty for the primary outcomes.

Outcomes	Number of participants (RCTs)	Eﬀect estimate (MD with 95% CI)	Certainty of the evidence
Pain severity/intensity (VAS scale) [25–27,29,30]	308 (5)	–1.12 [–2.16, –0.07]	⊕⊕⊝⊝ **Low**^a,b^
Pain intensity (NRS scale) [31]	107 (1)	–0.90 [–1.53, –0.27]	⊕⊕⊝⊝ **Low**^b,c^
Duration of pain (CMSS scale) [27]	50 (1)	–1.56 [–2.06, –1.06]	⊕⊕⊕⊝ **Moderate**^b^
Pain medication use (non-narcotic pain tablets) per week [29]	42 (1)	–0.10 [–3.39, 3.19]	⊕⊕⊕⊝ **moderate**^b^
**GRADE Working Group grades of evidence** High certainty: We are very confident that the true effect lies close to that of the estimate of the effect. Moderate certainty: We are moderately confident in the effect estimate. The true effect is likely to be close to the estimate of the effect, but there is a possibility that it is substantially different. Low certainty: Our confidence in the effect estimate is limited. The true effect may be substantially different from the estimate of the effect. Very low certainty: We have very little confidence in the effect estimate. The true effect is likely to be substantially different from the estimate of effect.			
**Explanation:** ^a^Inconsistency (high heterogeneity) ^b^Imprecision (small sample size) ^c^High risk of bias			

## Discussion

Summary of main findings vitamin D supplement for pain management in gynaecological conditions, especially primary dysmenorrhea and endometriosis, is not uncommon. A comprehensive search of the ICTRP identified seven registered RCTs on this topic – four on vitamin D for primary dysmenorrhea pain and, three on vitamin D for endometriosis pain.

All seven RCTs involved placebos. Six were conducted in Iran and one was conducted in the USA. This is not surprising given that Iran has a high prevalence of vitamin D deficiency [34]. The sample size was generally small, with an average of 36 participants per group. Four out of seven RCTs had a treatment duration of 8 or 12 weeks. Only two RCTs [26,30] had a follow-up period, which was two months post intervention.

The Endocrine Society clinical practice guideline [32] indicates the need for at least 1000 IU per day to raise the blood level of 25(OH)D to above 30 ng/mL and, for people with vitamin D deficiency, 50,000 IU once a week for eight weeks or its equivalent of 6000 IU daily to achieve a blood level of 25(OH)D above 30 ng/mL, followed by a maintenance dose of 1500–2000 IU daily. These doses and durations were largely observed in the included RCTs, although the doses, frequency of administration and duration were not consistently reported in the RCTs.

Several validated tools were used to measure pain, including VAS, CMSS and NRS. A statistically significant reduction in pain severity was observed in the vitamin D group when compared to placebo group in primary dysmenorrhea RCTs measuring VAS or NRS. The significant difference was not observed in the two endometriosis RCTs measuring VAS. In the context of endometriosis, statistically significant reduction in pain duration was observed when measured by CMSS.

In clinical research, the minimal clinically important difference (MCID) represents the smallest change to be considered clinically meaningful. Using VAS, a change of 1.0 cm is often considered MCID for chronic pain conditions, including endometriosis [35]. In this review, the reduction of 1.12 point based on five pooled studies can be considered MCID. However, using NRS, a minimum of a two-point reduction is required for an MCID. Therefore, the reduction of 0.90 point based on one RCT is not considered an MCID [36].

Pain medication use is another important measurement for pain research, though it is used with other outcome measures rather than alone [37]. Two RCTs [29,31] reported pain medication use, but there was either no significant difference between groups or the data reported restricted meta-analysis from being done.

Among the included RCTs, limited information about the safety profile of vitamin D was provided. When used within the maximum recommended dose, vitamin D is generally considered safe, though it is generally acknowledged that safety information is underreported in clinical studies [38].

### Comparison with previous systematic review research

The effect of vitamin D on primary dysmenorrhea or endometriosis has been evaluated in several recently published systematic reviews.

In a 2020 review [39] of 16 RCTs of various micronutrients for managing primary dysmenorrhea pain, three of the RCTs examined vitamin D. The authors concluded that pain severity was significantly decreased more in the vitamin D groups than placebo groups after two months of treatment, although the finding was not significant when the effect was evaluated one month post treatment.

Vitamin D for reducing pain in people with primary dysmenorrhea was also examined in a 2023 systematic review [22] of nine RCTs that focused on dose and treatment duration. The authors concluded that pain reduction was more obvious with an average weekly intake of over 50,000 IU vitamin D, over more or less than 70 days’ treatment duration and in any dose interval. These findings are consistent with this systematic review, although limited eligible RCTs restricts the review from exploring the correlation between the effects and dosage or treatment duration.

In contrast, a 2022 systematic review on four endometriosis RCTs did not find a significant effect of vitamin D on pain [23] when compared with placebo. This finding was confirmed by a 2023 systematic review [40] with five RCTs on the efficacy of vitamin D for pain management, for which the authors concluded that vitamin D’s effect was unclear across RCTs. These findings are consistent with this systematic review. Of note, all three registered endometriosis RCTs included in this systematic review were also included in the 2022 and 2023 systematic reviews.

### Limitations

This systematic review has several limitations. Six of the seven included RCTs were conducted in one country. Therefore, the included ethnic and demographic groups may not be diverse enough to make a general conclusion about the impact of vitamin D on primary dysmenorrhea or endometriosis. In addition, the included RCTs exhibited significant heterogeneity in terms of prescribed dosage of vitamin D and treatment duration. Small sample sizes may also impact the power to detect significant differences in pain outcomes. Concerns about risk of bias may influence the interpretation of the results and the overall quality of the evidence. The potential long-term safety of vitamin D supplement to manage pain were not adequately addressed, which may limit our understanding of the prolonged benefits and potential risks associated with long-term vitamin D use.

This systematic review was planned to include only RCTs registered in an ICTRP database. None of the seven included RCTs published a trial protocol.

### Implications for clinical practice

Studies have reported a positive association between vitamin D deficiency and menstrual dysfunction and pathogenesis [41], and an association between vitamin D deficiency and pain in people with primary dysmenorrhea or endometriosis [18]. When clinicians consider vitamin D supplement as a treatment option for people with primary dysmenorrhea, low or moderate certainty of evidence revealed from this systematic review should be taken into consideration. Future updates of clinical practice guidelines for primary dysmenorrhea and endometriosis considering evidence from available RCTs and systematic reviews are warrant.

### Implications for future research

Future RCTs should report any pharmaceutical interventions, particularly NSAIDs, and non-pharmaceutical interventions given to participants during the treatment and follow-up period. It is not uncommon for participants to take micronutrients and over-the-counter medicines for various purposes, but such information may not be voluntarily disclosed unless specifically asked and/or collected as part of the trial procedure.

The different phenotypes of endometriosis and baseline information about the serum 25[OH]D level should be stated. Together with detailed information on dosage, route of administration and treatment frequency and duration, this information enables subgroup analyses and contributes to better interpretation of RCT findings.

The potential risk of bias observed in this systematic review should be addressed in future studies. The most critical consideration is the application of intention-to-treat principle and the inclusion of all randomised participants in the final data analysis. Participant withdrawal and dropout, either voluntarily or terminated without specific reasons, should be documented and analysed. Participant compliance and non-compliance can also contribute to the validity of RCTs, so information about compliance should be included as potential confounding factors.

Overall, a high-quality RCT with proper statistical power that investigates vitamin D for primary dysmenorrhea and endometriosis pain management is needed to contribute to our understanding of the clinical implications of vitamin D for these conditions.

## Conclusion

This is the first systematic review evaluating the effect of vitamin D in pain management in two related gynaecological health conditions. It found a low certainty of evidence that vitamin D supplement could reduce pain severity or duration in people with primary dysmenorrhea. vitamin D’s benefits for endometriosis needs further exploration.

## Supporting information

S1 Table Numbered table of all studies identified in the literature search. (DOCX)

S2 Table Original searching result and exclusion code. (XLSV)

S3 Table RoB_2_Almassinokiani 2016. (DOCX)

S4 Table RoB_2_Amzajerdi 2023. (DOCX)

S5 Table RoB_2_Beh 2023. (DOCX)

S6 Table RoB_2_Mehdizadehkashi 2021. (DOCX)

S7 Table RoB_2_Nodler 2020. (DOCX)

S8 Table RoB_2_Pakniat 2019. (DOCX)

S9 Table RoB_2_Rahnemaei 2021. (DOCX)

S10 Table VD MS original data for meta and GRADE. (XLSV)

S11 Table PRISMA_2020_checklist_06052024. (DOCX)

## References

[1] Tsamantioti ESM. Heba StatPearls. Treasure Island, Florida: StatPearls Publishing; 2023.

[2] Ju H, Jones M, Mishra G. The prevalence and risk factors of dysmenorrhea. Epidemiol. Rev. 2014;36:104–13. 24284871 10.1093/epirev/mxt009

[3] Giudice LC, Kao LC. Endometriosis. Lancet. 2004;364(9447):1789–99. 15541453 10.1016/S0140-6736(04)17403-5

[4] Itani R, Soubra L, Karout S, Rahme D, Karout L, Khojah H. Primary dysmenorrhea: pathophysiology, diagnosis, and treatment updates. Korean J Fam Med. 2022;43(2):101–8. 35320895 10.4082/kjfm.21.0103 PMC8943241

[5] Bernardi M, Lazzeri L, Perelli F, Reis F, Petraglia F. Dysmenorrhea and related disorders. F1000Research. 2017;6:1645. 28944048 10.12688/f1000research.11682.1 PMC5585876

[6] Barcikowska Z, Rajkowska-Labon E, Grzybowska M, Hansdorfer-Korzon R, Zorena K. Inflammatory markers in dysmenorrhea and therapeutic options. International Journal of Environmental Research and Public Health. 2020;17(4). 10.3390/ijerph17041191 PMC7068519 32069859

[7] Karout S, Soubra L, Rahme D, Karout L, Khojah HMJ, Itani R. Prevalence, risk factors, and management practices of primary dysmenorrhea among young females. BMC Womens Health. 2021;21(1):392. doi: 10.1186/s12905-021-01532-w 34749716 PMC8576974

[8] Hailemeskel S, Demissie A, Assefa N. Primary dysmenorrhea magnitude, associated risk factors, and its effect on academic performance: evidence from female university students in Ethiopia. Int J Womens Health. 2016;8:489–96. doi: 10.2147/IJWH.S112768 27695366 PMC5034908

[9] Sourial S, Tempest N, Hapangama DK. Theories on the pathogenesis of endometriosis. Int J Reprod Med. 2014;2014:179515. doi: 10.1155/2014/179515 25763392 PMC4334056

[10] Dmowski WP, Ding J, Shen J, Rana N, Fernandez BB, Braun DP. Apoptosis in endometrial glandular and stromal cells in women with and without endometriosis. Hum Reprod. 2001;16(9):1802–8. doi: 10.1093/humrep/16.9.1802 11527879

[11] Wu Y, Strawn E, Basir Z, Halverson G, Guo S-W. Promoter hypermethylation of progesterone receptor isoform B (PR-B) in endometriosis. Epigenetics. 2006;1(2):106–11. doi: 10.4161/epi.1.2.2766 17965625

[12] Chantalat E, Valera M, Vaysse C, Noirrit E, Rusidze M, Weyl A. Estrogen receptors and endometriosis. International Journal of Molecular Sciences. 2020;21(8). 10.3390/ijms21082815 PMC7215544 32316608

[13] Herington JL, Bruner-Tran KL, Lucas JA, Osteen KG. Immune interactions in endometriosis. Expert Rev Clin Immunol. 2011;7(5):611–26. doi: 10.1586/eci.11.53 21895474 PMC3204940

[14] Welfare Ai. Endometriosis in Australia: prevalence and hospitalisations. Canberra: AIHW; 2019.

[15] Mardon AK, Leake HB, Hayles C, Henry ML, Neumann PB, Moseley GL, et al. The Efficacy of Self-Management Strategies for Females with Endometriosis: a Systematic Review. Reprod Sci. 2023;30(2):390–407. doi: 10.1007/s43032-022-00952-9 35488093 PMC9988721

[16] Burnett M, Lemyre M. No. 345-Primary Dysmenorrhea Consensus Guideline. J Obstet Gynaecol Can. 2017;39(7):585-95. 28625286 10.1016/j.jogc.2016.12.023

[17] Kalaitzopoulos DR, Samartzis N, Kolovos GN, Mareti E, Samartzis EP, Eberhard M, et al. Treatment of endometriosis: a review with comparison of 8 guidelines. BMC Womens Health. 2021;21(1):397. doi: 10.1186/s12905-021-01545-5 34844587 PMC8628449

[18] Anastasi E, Fuggetta E, De Vito C, Migliara G, Viggiani V, Manganaro L. Low levels of 25-OH vitamin D in women with endometriosis and associated pelvic pain. Clin Chem Lab Med. 2017;55(12):e282–4. 28453438 10.1515/cclm-2017-0016

[19] Giampaolino P, Della Corte L, Foreste V, Bifulco G. Is there a Relationship Between Vitamin D and Endometriosis? An Overview of the Literature. Curr Pharm Des. 2019;25(22):2421–7. doi: 10.2174/1381612825666190722095401 31333100

[20] Helde-Frankling M, Björkhem-Bergman L. Vitamin D in pain management. International Journal of Molecular Sciences. 2017;18(10) 10.3390/ijms18102170 PMC5666851 29057787

[21] Vallée A, Ceccaldi P, Carbonnel M, Feki A, Ayoubi J. Pollution and endometriosis: A deep dive into the environmental impacts on women’s health. BJOG. 2023;21. 10.1111/1471-0528.17687 37814514

[22] Chen Y-C, Chiang Y-F, Lin Y-J, Huang K-C, Chen H-Y, Hamdy NM, et al. Effect of Vitamin D Supplementation on Primary Dysmenorrhea: A Systematic Review and Meta-Analysis of Randomized Clinical Trials. Nutrients. 2023;15(13):2830. doi: 10.3390/nu15132830 37447156 PMC10343446

[23] Kalaitzopoulos DR, Samartzis N, Daniilidis A, Leeners B, Makieva S, Nirgianakis K, et al. Effects of vitamin D supplementation in endometriosis: a systematic review. Reprod Biol Endocrinol. 2022;20(1):176. doi: 10.1186/s12958-022-01051-9 36578019 PMC9795583

[24] Pazhohan A, Danaei-Mehrabad S, Mohamad-Rezaeii Z, Amidi F, Khodarahmian M, Shabani Nashtaei M, et al. The modulating effects of vitamin D on the activity of β-catenin in the endometrium of women with endometriosis: a randomized exploratory trial. Gynecol Endocrinol. 2021;37(3):278–82. doi: 10.1080/09513590.2020.1858780 33305626

[25] Almassinokiani F, Khodaverdi S, Solaymani-Dodaran M, Akbari P, Pazouki A. Effects of Vitamin D on Endometriosis-Related Pain: A Double-Blind Clinical Trial. Med Sci Monit. 2016;22:4960–6. doi: 10.12659/msm.901838 27986972 PMC5189720

[26] Amzajerdi AK, Ghorbali E, Pezaro S, Sarvi F. The effect of vitamin D on the severity of dysmenorrhea and menstrual blood loss: a randomized clinical trial. BMC Womens Health. 2023;23(1):138. 36973702 10.1186/s12905-023-02284-5 PMC10045437

[27] Behrouzi Lak T, Aghakhani N, Vahabzadeh D, Eghtedar S, Cheraghi R, Ghasemzadeh N, et al. A comparison of the effect of Vitamin D and Vitamin E supplementations, alone, and in combination, on reducing the intensity and duration of dysmenorrhea in women. Journal of Integrative Nursing. 2023;5(1):21–6. doi: 10.4103/jin.jin_49_22

[28] Mehdizadehkashi A, Rokhgireh S, Tahermanesh K, Eslahi N, Minaeian S, Samimi M. The effect of vitamin D supplementation on clinical symptoms and metabolic profiles in patients with endometriosis. Gynecol Endocrinol. 2021;37(7):640–5. doi: 10.1080/09513590.2021.1878138 33508990

[29] Nodler JL, DiVasta AD, Vitonis AF, Karevicius S, Malsch M, Sarda V, et al. Supplementation with vitamin D or ω-3 fatty acids in adolescent girls and young women with endometriosis (SAGE): a double-blind, randomized, placebo-controlled trial. Am J Clin Nutr. 2020;112(1):229–36. doi: 10.1093/ajcn/nqaa096 32453393 PMC7326593

[30] Pakniat H, Chegini V, Ranjkesh F, Hosseini MA. Comparison of the effect of vitamin E, vitamin D and ginger on the severity of primary dysmenorrhea: a single-blind clinical trial. Obstet Gynecol Sci. 2019;62(6):462–8. doi: 10.5468/ogs.2019.62.6.462 31777743 PMC6856484

[31] Rahnemaei FA, Gholamrezaei A, Afrakhteh M, Zayeri F, Vafa MR, Rashidi A, et al. Vitamin D supplementation for primary dysmenorrhea: a double-blind, randomized, placebo-controlled trial. Obstet Gynecol Sci. 2021;64(4):353–63. doi: 10.5468/ogs.20316 34010550 PMC8290151

[32] Holick M, Binkley N, Bischoff-Ferrari H, Gordon C, Hanley D, Heaney R, et al. Evaluation, treatment, and prevention of vitamin D deficiency: an Endocrine Society clinical practice guideline. J Clin Endocrinol Metab. 2011;96(7):1911–30. 21646368 10.1210/jc.2011-0385

[33] Delgado DA, Lambert BS, Boutris N, McCulloch PC, Robbins AB, Moreno MR, et al. Validation of Digital Visual Analog Scale Pain Scoring With a Traditional Paper-based Visual Analog Scale in Adults. J Am Acad Orthop Surg Glob Res Rev. 2018;2(3):e088. doi: 10.5435/JAAOSGlobal-D-17-00088 30211382 PMC6132313

[34] Aghapour B, Kheirouri S, Alizadeh M, Khodayari-Zarnaq R. Vitamin D deficiency prevention policies in Iran: a retrospective policy analysis. Frontiers in Nutrition. 2023;10:1249402. 37680901 10.3389/fnut.2023.1249402 PMC10482268

[35] Gerlinger C, Schumacher U, Faustmann T, Colligs A, Schmitz H, Seitz C. Defining a minimal clinically important difference for endometriosis-associated pelvic pain measured on a visual analog scale: analyses of two placebo-controlled, randomized trials. Health Qual Life Outcomes. 2010;8:138. doi: 10.1186/1477-7525-8-138 21106059 PMC3002916

[36] Kohli N, Jarnagin B, Stoehr AR, Lamvu G. An observational cohort study of pelvic floor photobiomodulation for treatment of chronic pelvic pain. J Comp Eff Res. 2021;10(17):1291–9. doi: 10.2217/cer-2021-0187 34490787

[37] Nawai A, Leveille SG, Shmerling RH, van der Leeuw G, Bean JF. Pain severity and pharmacologic pain management among community-living older adults: the MOBILIZE Boston study. Aging Clin Exp Res. 2017;29(6):1139–47. doi: 10.1007/s40520-016-0700-9 28224474 PMC5565717

[38] Maggini V, Crescioli G, Ippoliti I, Gallo E, Menniti-Ippolito F, Chiaravalloti A, et al. Safety profile of vitamin D in Italy: An analysis of spontaneous reports of adverse reactions related to drugs and food supplements. J Clin Med. 2023;12(14). 10.3390/jcm12144726 PMC10381134 37510843

[39] Saei Ghare Naz M, Kiani Z, Rashidi Fakari F, Ghasemi V, Abed M, Ozgoli G. The Effect of Micronutrients on Pain Management of Primary Dysmenorrhea: a Systematic Review and Meta-Analysis. J Caring Sci. 2020;9(1):47–56. doi: 10.34172/jcs.2020.008 32296659 PMC7146731

[40] Kahlon BK, Simon-Collins M, Nylander E, Segars J, Singh B. A systematic review of vitamin D and endometriosis: role in pathophysiology, diagnosis, treatment, and prevention. F&S Reviews. 2023;4(1):1–14. doi: 10.1016/j.xfnr.2022.11.005

[41] Bashmakova NV, Lisovskaya TV, Vlasova VY. Pathogenetic role of vitamin D deficiency in the development of menstrual dysfunction in pubertal girls: a literature review. Gynecol Endocrinol. 2017;33(sup1):52–5. doi: 10.1080/09513590.2017.1404235 29264978

