# Supplementary material for: Vitamin D for primary dysmenorrhea and endometriosis-related pain – A systematic review of registered RCTs
Source: PLoS One. 2025 Apr 21;20(4):e0321393. doi: 10.1371/journal.pone.0321393 (PMC12011270; doi:10.1371/journal.pone.0321393)
Supplement: S1 Table — (DOCX) [file pone.0321393.s001.docx]

**Supplementary Table 1: Numbered table of all studies identified in the literature search**

| **No.** | **Ref** | **Full title** | **Included/excluded & Reason for exclusion** |
| --- | --- | --- | --- |
| 1 | 24 | Pazhohan A, Danaei-Mehrabad S, Mohamad-Rezaeii Z, Amidi F, Khodarahmian M, Shabani Nashtaei M, et al. The modulating effects of vitamin D on the activity of β-catenin in the endometrium of women with endometriosis: a randomized exploratory trial. Gynecol Endocrinol. 2021;37(3):278-82. | ***Excluded, study did not assess pain-related outcomes*** |
| 2 | 25 | Almassinokiani F, Khodaverdi S, Solaymani-Dodaran M, Akbari P, Pazouki A. Effects of Vitamin D on Endometriosis-Related Pain: A Double-Blind Clinical Trial. Med Sci Monit. 2016;22:4960-6. | **Included** |
| 3 | 26 | Amzajerdi Azam KM, Ghorbali Elham, Pezaro Sally, Sarvi Fatemeh The effect of vitamin D on the severity of dysmenorrhea and menstrual blood loss: a randomized clinical trial. BMC Womens Health. 2023 Mar 27;23(1):138. | **Included** |
| 4 | 27 | Behrouzi-Lak Tahereh AN, Davoud Vahabzadeh, Samereh Eghtedar, Rozita Cheraghi, Nazafarin Ghasemzadeh, Vahid Alinejad, Maryam Mesgarzadeh. A comparison of the effect of Vitamin D and Vitamin E supplementations, alone, and in combination, on reducing the intensity and duration of dysmenorrhea in women: A randomized controlled trial. Journal of Integrative Nursing. 2023;5(1):21-6. | **Included** |
| 5 | 28 | Mehdizadehkashi A, Rokhgireh S, Tahermanesh K, Eslahi N, Minaeian S, Samimi M. The effect of vitamin D supplementation on clinical symptoms and metabolic profiles in patients with endometriosis. Gynecol Endocrinol. 2021;37(7):640-5. | **Included** |
| 6 | 29 | Nodler JL, DiVasta AD, Vitonis AF, Karevicius S, Malsch M, Sarda V, et al. Supplementation with vitamin D or ω-3 fatty acids in adolescent girls and young women with endometriosis (SAGE): a double-blind, randomized, placebo-controlled trial. Am J Clin Nutr. 2020;112(1):229-36. | **Included** |
| 7 | 30 | Pakniat H, Chegini V, Ranjkesh F, Hosseini MA. Comparison of the effect of vitamin E, vitamin D and ginger on the severity of primary dysmenorrhea: a single-blind clinical trial. Obstet Gynecol Sci. 2019;62(6):462-8. | **Included** |
| 8 | 31 | Rahnemaei FA, Gholamrezaei A, Afrakhteh M, Zayeri F, Vafa MR, Rashidi A, et al. Vitamin D supplementation for primary dysmenorrhea: a double-blind, randomized, placebo-controlled trial. Obstet Gynecol Sci. 2021;64(4):353-63. | **Included** |
